# The Influence of Affective Feedback Adaptive Learning System on Learning Engagement and Self-Directed Learning

**DOI:** 10.3389/fpsyg.2022.858411

**Published:** 2022-04-27

**Authors:** Hsin-Lan Liu, Tao-Hua Wang, Hao-Chiang Koong Lin, Chin-Feng Lai, Yueh-Min Huang

**Affiliations:** ^1^Department of Engineering Science, National Cheng Kung University, Tainan, Taiwan; ^2^Department of Information and Learning Technology, National University of Tainan, Tainan, Taiwan

**Keywords:** affective computing, adaptive learning system, AEQ learning emotions, learning engagement, self-directed learning

## Abstract

The outbreak of the two-year corona virus has made a great difference on existing methods of learning and instruction. Online education has become a crucial role to maintain non-stop learning after the post-epidemic period. The advanced technologies and growing popularity of network equipment have made it easy to deploy remote connections. However, teachers still face challenges when they actually implement distance courses. During the learning process, the quality of learning can be improved if the researchers consider multiple factors, including emotions, attitudes, engagement, cognition, neuroscientific and cultural psychology. After analyzing these factors, instructors can have better understanding of students’ mental building and cognitive understanding in their process of learning, and be familiar with the way of interaction with students and appropriately adjust their teaching. Therefore, the current study established a learning system that aimed to understand learners’ emotional signals during learning by applying the adaptive-feedback emotional computing technology. The purpose of the system was to allow learners to (1) self-examine their learning condition, (2) enhance their self-directed learning, (3) help learners who are in negative learning emotions or settings to lower anxieties, and (4) promote their learning attitudes and engagement. Result showed that the system with the adaptive-feedback emotional computing technology has significantly improved the learning effectiveness, lowered learning anxieties and increased students’ self-directed learning.

## Introduction

Owing to the unusual outbreak of COVID-19 in 2020, staying in a house without going public has become an essential place for people to avoid mass gatherings and the propagation of the virus. Distance learning and stay-at-home work were unexpectedly developed rapidly. Many challenges emerge when both students and teachers conduct distance learning. How to adjust learning materials or tasks to accommodate students’ conditions is necessarily stated. Alhumaid et al. (2020) stated the importance of course designers to design distance learning lessons (Roman and Plopeanu, 2021). Without carefully designing the distance learning, it will have a negative impact on students’ learning effectiveness and their engagement (Aboagye, 2021; Aboagye et al., 2021). Instructors hardly examine students’ learning progress and problems when students are involved in the learning platform. Although many distance learning systems adopt the assessment method to evaluate students’ learning conditions, teachers are not able to predict potential problems or provide proper guidance when students sitting in front of computers become distracted by other things. Distance learning differs from the face-to-face classroom learning. Students’ learning emotions or anxieties in their current situations cannot be directly perceived by instructors to provide appropriate interventions or support students’ needs (Daouas and Lejmi, 2018; Al-Fraihat et al., 2020). Given the fact that interaction between teachers and students greatly affects students’ learning, and establishing a positive classroom atmosphere is also a key to successful learning in school (Sun et al., 2019; Aktekin and Celebi, 2020). Recent research on neuroscientific foundations of learning has created major changes in both methods and theories about the study of learning and the brain, leading to better applicability of brain findings to educational issues and questions. As individual culture varies, various neural responses and multiple emotions present. Learning is interwoven with the factors like mental state, emotion, cognition, and neurology. These factors are associated with individuals’ learning effectiveness and their understanding of the learning material during their cognitive processing. As stated above, instructional design should provide proper interventions or support students’ needs according to individual differences in the distance learning. The current study established an adaptive learning system that employed affective computing technology with the emotional feedback mechanism to support students’ online courses. The system was to give adaptive feedback by analyzing students’ affective conditions when they were engaged in the learning system. By implementing adaptive feedback in the online course, it is possible to reinforce students’ self-directed learning ability, adjust their negative emotions, reduce their learning anxieties, and improve their learning attitude and engagement after receiving adaptive feedback.

## Literature Review

### Learning and Learning Emotion

Emotion enriches our lives, and it is also considered as a crucial issue affecting individuals’ learning achievement (Quinlan, 2016; Zembylas and Schutz, 2016; Hill et al., 2021). The positive learning emotion corresponds to more efforts among individuals who are willing to participate in learning (Liu et al., 2018; Molinillo et al., 2018).

Students tend to understand their goals, maintain their motivation and desire to persist without easily giving up if they have positive learning emotions. Students tend to lose their interests and are unwilling to engage in learning if their emotions show fear, anxieties and worries in the learning system. While students experience a high level of anxiety in the learning system, they could demotivate their learning. It is important to consider the aspects of anxiety and learning attitudes as they play an essential part in supporting learning (Endler and Kocovski, 2001; Ali and Anwar, 2021; Wang et al., 2021).

Learning anxiety refers to negative emotions that occur during learning, such as fear, worries, and stress. Many studies have discussed the issue of how anxiety affects students’ ability to learn subjects like language, mathematics, and science. They also explored that different levels of anxiety among students would result in different learning outcomes. According to the findings from Sun et al. (2017), appropriate anxiety has a positive effect on promoting learning effectiveness, whereas overwhelming anxiety that students experience can negatively intervene competence performance and learning achievement (Sun et al., 2017).

Anxiety can be a crucial factor influencing the test scores, but it may be the reason that affects students to have low scores of learning achievement (Mandler and Sarason, 1952; Hyseni Duraku and Hoxha, 2018). Research has indicated that learning achievement has a strong relationship among variables involved in the learning system. Learning anxiety has a negative relationship with learning achievement, whereas learning attitude has a positive relationship with learning motivation (Hong-Sheng, 2005).

Students with high levels of anxiety might be likely to do more exercises, and have a relationship with students who are with low levels of anxiety. Students with low levels of anxiety might be highly motivated to take part in learning, and their learning strategies show a strong relationship with their learning achievement (Warr and Downing, 2000; Bai et al., 2020).

### Learning Engagement

Learning engagement refers to individuals’ behavioral conditions actively participating in the learning activity. It involves the degree of learning motivation, learning process and self expectation (Chen, 2017; Jung and Lee, 2018). Roberts and McNeese (2010) stated that learning engagement emphasizes learning activities that support students to have successful experience in overcoming difficulties. It has a relationship between students’ learning behaviors and learning environment.

Fredricks et al. (2016) proposed three dimensions of different relationships in the learning engagement, including behavioral engagement, motional engagement, and cognitive engagement. Chen et al. (2018) conducted a study on exploring whether a game-based learning supported students’ learning engagement in the flipped-learning model. Results indicated that the game-based learning assisted flipped-learning students in improving their learning scores and producing better learning and achievement.

Lai et al. (2019) investigated whether social groups engaged in a community of practice would affect students’ learning achievement and engagement. The students in the experimental groups used both the online English language learning system and the social group establishment, whereas the students in the control group merely accessed the online English language learning system. Results showed that those students who were involved in two modes of learning (learning system and social group) showed a high level of interaction among others, compared to the students who were involved in only one mode of learning (learning system). However, there was a significant difference in learning achievement and engagement between two groups.

The current study aimed to investigate students’ learning achievement and learning engagement after they participated in the learning system with adaptive emotional feedback mechanism. The relationship among the variables of learning engagement, achievement and emotions were also explored.

### Self-Directed Learning

Tough (1978) studied on the self-directed learning focusing learning project as a unit to evaluate self-directed learning ability. The unit included a series of learning activities with an overall duration of 7 h or above every day. It must do it in a way that promotes specific knowledge or self-education and prepares themselves for sustaining the development of life-long learning behavior.

During learning, self-directed learners are mostly likely to identify their own learning needs, determine their goals, and reach the destination. They can work independently by planning their learning steps, diagnosing their own needs, establishing their learning strategies, and be responsible for their learning and development. With or without support or assistance obtained from any group, they can effectively take control over their learning and satisfy their self-requirements.

Researchers have pointed out that self-directed learners refer to individuals who are able to diagnose their personal needs, build their goals, and sustain their learning practices by applying proper learning strategies after locating learning resources. They then can achieve optimal learning outcomes (Beckers et al., 2016; Rashid and Asghar, 2016).

In terms of the exploration of digital self-directed learning, more recent studies have found that there exists the relationship between self-directed learning ability and digital learning ability, and they have further explored learners’ motivation for using digital technology to facilitate learning (Lai and Wang, 2017; Eroglu and Ozbek, 2018; Angriani and Nurcahyo, 2019; Chau et al., 2021).

The educational learning environment and contexts have dramatically changed. Self-directed learning and digital learning have a strong relationship with learning motivation, while learning motivation has a relationship with learning achievement (Geng et al., 2019). In the current study, the developed adaptive emotional feedback system was expected to effectively facilitate student learning, and toward the improvement of self-directed learning and learning achievement.

## Research Method

The established system of adaptive emotional feedback contained two modes of collecting participants’ emotions, including emotional recognition to capture facial emotion and text recognition to recognize semantic emotion. The collected data after recognition will be recorded to the database of learning conditions.

The system sent the data to the affective agents that corresponded to the users with relevant emotional reactions and appropriate feedback throughout their learning. The learning zones offered related videos and documents of the technology and art that allowed users to switch. Meanwhile, the system offered a self-quiz function and recorded the learning progress, which was designed to scaffold students’ learning during their engagement with the course learning activities. The system was developed using the web-based format. Users can easily access the system easily.

The system development tool: The client used HTML, CSS, and JavaScript to build webpage services as the front end, while the server adopted MySQL with PHP code as the back end. The development of emotion recognition was developed using the kit of facial analysis provided by the Clmtrackr program. The semantic analysis was built by Python as the interface for data processing and the back-end server. The built-in computer camera system on computers was taken to detect users’ facial emotion.

Many students have experienced boring or uninterested learning materials from either accessing pure texts or teaching videos (Greenagel, 2002). When students have negative emotions during their involvement in digital learning, they will inevitably generate negative impact on their motivation, willingness and effectiveness in learning. It would necessarily include a motivated scenario or play to transform students’ negative emotions, thereby attracting students to sustain their learning (Brown and Voltz, 2005). The study provided adaptive feedback mechanisms in the learning system to facilitate students’ learning effectiveness after detecting users’ emotions. The current study built an adaptive learning system with emotional feedback mechanism to construct an online course of technology and art, by applying the adaptive presentation technology.

**FIGURE 1:**
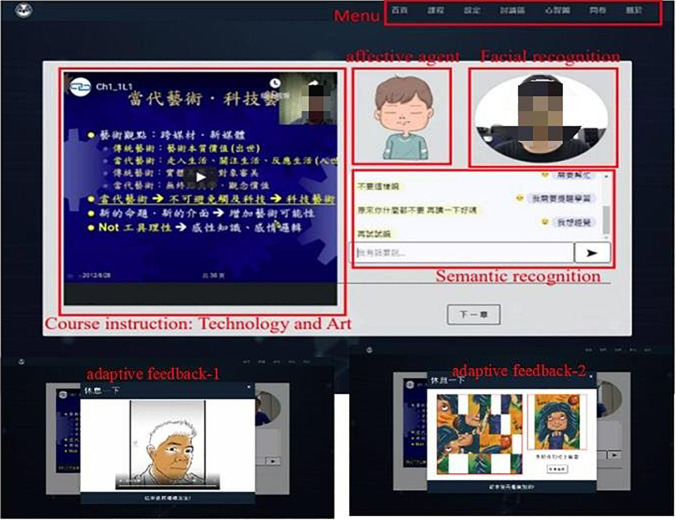
The affective feedback adaptive learning system.

The system identified students’ emotions by constructing both facial and semantic recognitions. While users accessed online digital learning materials, the system automatically detected users’ emotions and sent feedback messages by the affective agents. Figure 1 detected users’ emotions and gave feedback after a while in order to avoid distracting users’ learning. The semantic recognition is part of immediate feedback generated by Chatbot after the users actively chatted with Chatbot. Chatbot will give certain encouragement and praise (see Figure 1).

**FIGURE 2:**
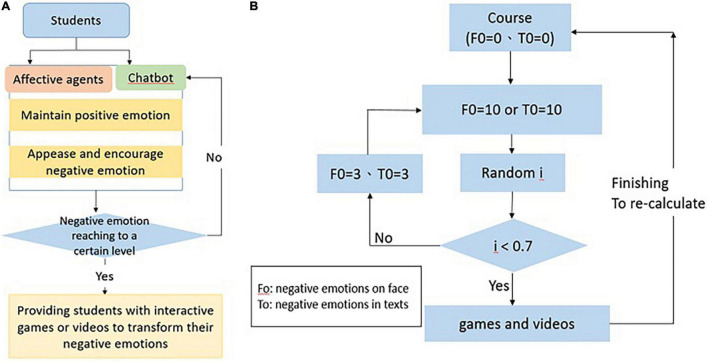
**(A,B)** The system algorithm architecture.

During learning, students might lose interest or feel bored over a long period of time. The system accumulated the final data by collecting comprehensive negative emotions of students from facial and semantic recognitions. When the collected data reached a certain level (the threshold), the system provided students with the adaptive feedback relevant to interactive games or videos. After accessing different learning content, the system aimed to transform students’ negative emotions, motivate their learning and thereby improve their learning achievement. While giving feedback, the system offered choices for learners to select their intended feedback (see Figure 2A). The system re-calculated a collection of negative emotions once the feedback has been given out. The algorithm is shown in the Figure 2B.

## Participants and Procedures

**FIGURE 3:**
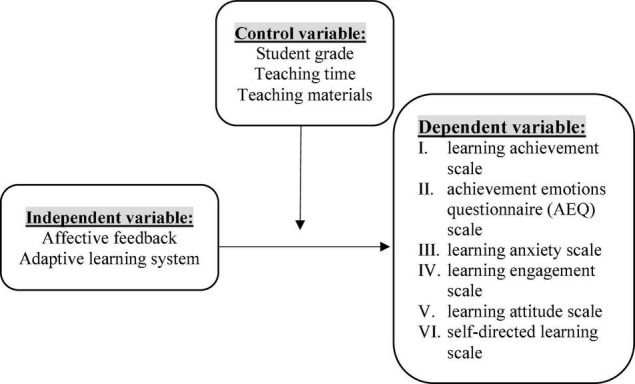
A research flowchart.

A total of 70 university students from southern Taiwan participated in this study, and were divided into two groups with 35 students (odd number) for the control group and 35 students (plural number) for the experimental group. None of the students had any experience with adaptive feedback learning systems. Both groups of students accessed learning material at home. Each student was confirmed to have a webcam and internet connections. A 6-week period of experiment was planned using the distance learning instruction. To ensure students’ equivalent learning background, the pre-test of learning achievement was distributed, along with the questionnaires of achievement emotions questionnaire (AEQ), learning anxiety, learning engagement, learning attitude and self-directed learning scale. The study used presentation video to introduce the purpose of the course and the adaptive feedback mechanism, and ensured students’ operations of the system after completing the introduction. Students independently assessed the learning material without any limitation. At the end of the course, they were invited to complete the post-test and post-questionnaires (see Figure 3).

## Results

This study adopted a quasi-experimental method, and used the SPSS 21.0 software to carry out the statistical analysis. The specific methods included one-way ANCOVA and independent sample *t*-test.

In terms of learning achievement, a pre- and post-test, with a total score of 100 points, was constructed based on the content generated by an experienced Technology and Art teacher. The study conducted a one-way ANCOVA analysis, using the pretest as the covariance, post-test as the dependent variable, different teaching approaches as the independent variable, and different groups of students as the independent samples. Results showed that the students in the experimental group (*M* = 83.28, SD = 14.59) had significantly better learning effectiveness (*F* = 5.64, *p* = 0.002 < 0.05) than those in the control group (*M* = 73.14, *SD* = 22.46), showing that the learning effectiveness showed the significant difference between two groups when the student accessed the different learning approaches.

The AEQ was adapted by Pekrun et al.’s (2005) AEQ, with two dimensions of before study and after study, a 5-point Likert scale (*1 = strongly disagree; 5 = strongly agree*). The AEQ consisted of 30 items, including pleasure, hope, pride, anger, anxiety, humiliation, frustration, and boredom. The study used all items to explore users’ achievement emotions. Table 1 shows the result of the analysis of the AEQ using an independent sample *t*-test. It is found that the type of pleasure of the emotion in the experimental group was significantly better than that of the control group. The types of anxiety and humiliation of the emotion in the control group was significantly better than those of the experimental group.

**TABLE 1:** The AEQ independent sample *T*-test results between two groups.

Types	M	*t*	*p*
Learning emotion –Pleasure	3.70	2.034	0.046
Learning emotion –hope	3.71	0.942	0.350
Learning emotion –pride	3.49	1.558	0.124
Learning emotion –anger	3.43	1.268	0.209
Learning emotion –anxiety	3.17	2.108	0.039
Learning emotion –humiliation	3.02	2.003	0.049
Learning emotion –frustration	3.24	1.730	0.088
Learning emotion -boredom	3.24	1.262	0.211

In terms of learning anxiety, the study adapted Venkatesh’s (2000) scale, with a 5-point Likert scale (*1 = strongly disagree; 5 = strongly agree*) to evaluate the students’ attitudes of logical thinking, control and debugging, to examine students’ learning anxiety after using the learning system. The analysis of one-way ANCOVA indicated that there was significant difference (*F* = 5.62, *p* = 0.02 < 0.05) between two learning approaches when students participated in different learning approaches. The students in the experimental group (*Adjusted Mean* = 3.03, SD = 0.05) with the adaptive feedback mechanism had significantly lower anxiety than those in the control group (*Adjusted Mean* = 3.22, SD = 0.05) without adaptive feedback mechanism.

The learning engagement scale was modified from several different learning scales, including Fredricks et al.’s (2004) categorization of learning engagement studies, Kong et al.’s (2003) student engagement scale in mathematics, with a 5-point Likert scale (*1 = strongly disagree; 5 = strongly agree*), to evaluate students’ learning engagement after using the learning system. The analysis of one-way ANCOVA indicated that there was significant difference (*F* = 5.18, *p* = 0.02 < 0.05) between two groups when students participated in different learning approaches. The students in the experimental group (*Adjusted Mean* = 3.79, SD = 0.09) with the adaptive feedback mechanism had significantly better learning engagement than those in the control group (*Adjusted Mean* = 3.50, SD = 0.09) without the adaptive feedback mechanism. The learning engagement of the students in the adaptive feedback learning system showed better than those in the non-adaptive feedback learning system.

In addition, four dimensions of learning engagement were further explored, in terms of skills, performance, attitude and interaction with the learning system. A significant difference was found between two groups in the two dimensions of learning attitude, and interaction with the system. The learning engagement of students in the aspects of attitudes and interaction with the system in the experimental group was significantly better than that of students in the control group (see Table 2).

**TABLE 2:** Learning engagement of homogeneity of regression coefficients and the analysis of covariance.

	The homogeneity of regression coefficients		Covariance		Adjusted mean	
Items	Source of variation	*p*	*F*	*p*	Experimental Group (*N* = 35)	Control Group (*N* = 35)
Skills	Group and pre-test	0.313	2.260	0.137	3.75	3.54
Performance	Group and pre-test	0.359	3.771	0.056	3.84	3.56
Learning attitude	Group and pre-test	0.822	7.25*	0.009	3.78	3.42
Interaction with the system	Group and pre-test	0.594	5.29*	0.025	3.75	3.43

The learning attitude was modified from Sung et al. (2015) learning attitude scales, with a 5-point Likert scale (*1 = strongly disagree; 5 = strongly agree*), to investigate students’ attitudes toward the engagement of the learning system. The analysis of one-way ANCOVA revealed that there was significant difference (*F* = 6.00, *p* = 0.02 < 0.05) between two groups when students participated in different learning approaches. The students in the experimental group (*Adjusted Mean* = 3.56, SD = 0.07) with the adaptive feedback mechanism had significantly better learning attitudes than those in the control group (*Adjusted Mean* = 3.50, SD = 0.07) without the adaptive feedback mechanism. The learning attitudes of the students in the adaptive feedback learning system demonstrated better than those in the non-adaptive feedback learning system.

The self-directed learning was modified from Guglielmino’s (1977) learning attitude scales, with a 5-point Likert scale (*1 = strongly disagree; 5 = strongly agree*), to examine students’ self-directed learning toward the use of the learning system. The analysis of one-way ANCOVA revealed that there was significant difference (*F* = 6.27, *p* = 0.01 < 0.05) between two learning approaches when students participated in different learning approaches. The students in the experimental group (*Adjusted Mean* = 3.71, SD = 0.06) with the adaptive feedback mechanism had significantly better self-directed learning than those in the control group (*Adjusted Mean* = 3.46, SD = 0.06) without adaptive feedback mechanism. The self-directed learning of students in the adaptive feedback learning system demonstrated better than those in the non-adaptive feedback learning system.

## Correlation Coefficients Between the Different Variables

The correlation coefficients among the variables (learning achievement, AEQ, learning anxiety, learning engagement, learning attitude, and self-directed learning) was further discussed to investigate the relationships between them, including learning achievement, learning emotion, learning anxiety, learning engagement, learning attitude and self-directed learning. Table 3 shows the result. It was found that while the students’ learning achievement had a positive correlation with AEQ [*r(70)* = 0.25, *p* = 0.037], learning engagement [*r(70)* = 0.27, *p* = 0.02], learning attitude [*r(70)* = 0.26, *p* = 0.028], and self-directed learning [*r(70)* = 0.24, *p* = 0.04], it had a negative correction with learning anxiety [*r(70)* = −0.23, *p* = 0.047]. While the AEQ had a positive correlation with learning engagement [*r(70)* = 0.23, *p* = 0.047], learning attitude [*r(70)* = 0.36, *p* = 0.002], and self-directed learning [*r(70)* = 0.25, *p* = 0.035], it had a negative correction with learning anxiety [*r(70)* = −0.28, *p* = 0.018]. The learning engagement had a positive correlation with learning attitude [*r(70)* = 0.87, *p* = 0.000], whereas it had a negative correlation with self-directed learning [*r(70)* = −0.77, *p* = 0.000]. The learning anxiety had a negative correction with self-directed learning [*r(70)* = −0.23, *p* = 0.047], whereas the self-directed learning had a positive correction with learning attitude [*r(70)* = 0.65, *p* = 0.000]. There was no significant relationship between learning anxiety and learning engagement.

**TABLE 3:** Pearson correlation analysis of different variables.

	1	2	3	4	5	6
Learning achievement	−					
Learning emotion	0.25*	−				
Learning anxiety	−0.23*	−0.28*	−			
Learning engagement	0.27*	0.23*	–0.14	–		
Learning attitude	0.26*	0.36**	–0.19	0.87**	–	
Self-directed learning	0.24*	0.25*	−0.23*	0.77**	0.65**	–

*p < 0.05, **p < 0.01, ***p < 0.001.

From the above analysis, it revealed that positive learning emotion, learning anxiety, engagement, attitudes, and self-directed learning can all be attributed to the reasons of the promotion of learning achievement after using the learning system. Students with greater positive emotions can improve their learning engagement, attitudes and self-directed learning ability while reducing their learning anxieties. Therefore, the engagement of the adaptive feedback learning system enables them to demonstrate self-directed learning that was involved in active and self-disciplined learning in distance learning activities although potential learning anxiety existed. Meanwhile, the positive learning emotions can enhance learning engagement and attitudes, and thereby promoting learning greater learning attitudes after eagerly engaging the learning activities. Concisely, students can effectively adapt distance learning and become active learners if they have positive emotions, eager engagement, positive attitudes, self-directed learning abilities, and low anxiety in the learning process.

## Conclusion

The study has presented that the students of the experimental group performed better than those of the control group in the aspects of learning achievement, AEQ (learning emotion), learning anxiety, learning engagement, learning attitude, and self-directed learning. According to the findings, the designed learning system supported students in friendly engaging their learning. The system accords with the study conducted by Lin et al. (2015) that assisted students in cognitive development through the adaptive emotional feedback mechanism.

In addition, few studies investigated learning effectiveness that tailored the adapted emotional feedback mechanisms embedded in the Technology and Art learning contexts and used the recognition-supported techniques with both semantics and emotions to support distance learning. Many studies successfully engaged students in learning but they merely included semantic recognition without considering both recognition techniques of semantics and emotion. The current study applied recognition techniques of semantics and emotions through the accumulation of negative emotions using algorithms. Whenever the system recognized users’ negative emotions (boredom or tiredness), it gave immediate feedback or transformed users’ negative emotions to sustain learning and give alternative choices of learning materials throughout the process. The design of the current system proved the needs to design adaptive emotional feedback catered for users’ learning in distance learning contexts.

The positive learning emotion has a positive impact on self-directed learning, which also proved McCombs and Whisler’s (1989) studies on evidencing the relationship among learning anxiety, learning engagement, learning attitude, and self-directed learning ability. The findings on learning engagement echoes Rashid and Asghar (2016) studies on the impact of learning engagement and self-directed learning ability; it suggested that self-directed learning manifests learners’ independent learning, and thereby it supports positive learning attitudes. As for the relationship on learning anxiety, Uzun (2016) stated that there was no significant relationship between foreign language learning anxiety and self-directed learning ability. Different from Uzun’s (2016) findings, the current study proved its value with learning anxiety in the distance learning contexts, as appropriate learning anxiety manifested students’ values on the target subject itself. In the current study, the positive learning attitude enhanced students’ independent learning ability. It would also increase learning engagement and attitudes, and indirectly reduce learning anxiety. No significant relationship was found among learning anxiety, learning engagement and learning attitudes. The study confirmed these variables in their indirect relationship when all the dependent variables were mutually related.

Many platforms emphasize learning effectiveness throughout the process, and some of which included digital game-based learning and the evaluation. However, few studies take emotional feedback into consideration to enhance students’ engagement and learning.

The study aimed to promote students’ participation, increase self-directed learning ability and reduce learning anxieties throughout the learning process in the proposed adaptive emotional feedback learning system. Other digital learning systems can simulate schools’ learning settings and processes, but they cannot offer insight into individuals’ complex emotions and give appropriate feedback. Unlike the authentic learning environment (classroom), teachers can adjust instructional methods and offer appropriate materials for students when they perceive students’ negative emotions and demotivate learning behaviors. The general learning system fails to meet the needs of detecting individuals’ emotional obstruction and to give proper feedback. This would not only have a negative impact on learning achievement, learning engagement, but also increase learning anxiety and weaken the students’ ability to self-directed learning. Conversely, the current study provided adaptive feedback whenever it recognized users’ negative emotions from detecting their semantics and emotions in the proposed adaptive emotional feedback system. It scaffolded students’ learning in facilitating their engagement of online learning and adjusted their negative emotions in the learning process, thereby greatly strengthening self-directed learning ability.

## Data Availability Statement

The original contributions presented in the study are included in the article/supplementary material, further inquiries can be directed to the corresponding author.

## Author Contributions

H-CL, C-FL, and Y-MH: study conception and design and writing sections of the manuscript. H-LL and T-HW: data collection, analysis and interpretation of results, and writing the first draft of the manuscript. C-FL and Y-MH: financial support. All authors contributed to the article and approved the submitted version.

## Conflict of Interest

The authors declare that the research was conducted in the absence of any commercial or financial relationships that could be construed as a potential conflict of interest.

## Publisher’s Note

All claims expressed in this article are solely those of the authors and do not necessarily represent those of their affiliated organizations, or those of the publisher, the editors and the reviewers. Any product that may be evaluated in this article, or claim that may be made by its manufacturer, is not guaranteed or endorsed by the publisher.
